# Understanding correlates of infant mortality in Ethiopia using 2019 Ethiopian mini demographic and health survey data

**DOI:** 10.1097/MS9.0000000000000629

**Published:** 2023-04-12

**Authors:** Kebede Lulu Adebe, Senahara Korsa Wake, Sagni Daraje Yadata, Ketema Bedane Gondol, Gizachew Gobebo Mekebo, Temesgen Senbeto Wolde, Terefa Bechera, Belema Hailu Regesa, Agassa Galdassa, Kumera Dereje Yadata

**Affiliations:** a Department of Statistics, College of Natural and Computational Sciences, Ambo University, Ambo; b Department of Statistics, College of Natural and Computational Science, Jimma University, Jimma, Ethiopia

**Keywords:** 2019 EMDHS, correlates, cox proportional hazard model, ethiopia, infant mortality

## Abstract

Infant mortality is one of the most sensitive and commonly used indicators of the social and economic development of a nation. Ethiopia is among the African countries with high infant mortality rates. This study aimed to understand and identify correlates of infant mortality in Ethiopia.

**Methods:**

The data, used in this study, were drawn from 2019 Ethiopian Demographic and Health Survey data. The multivariable Cox proportional hazard analysis was done to identify the correlates of infant mortality.

**Results:**

Infant mortality rate was high in the earlier age of months. Males, higher birth order and rural residences were at higher risk of dying before first birthday compared with respective reference groups whereas health facility deliveries, single births, rich wealth indices and older maternal age were at lower risk of dying before first birthday compared with respective reference groups.

**Conclusion:**

The study found that age of mother, place of residence, wealth index, birth order, type of birth, child sex and place of delivery were statistically significant in affecting the survival of the infants. Thus, health facility deliveries should be encouraged and multiple birth infants should be given special care. Furthermore, younger mothers should better care of their babies to improve the survival of infants in Ethiopia.

## Introduction

Highlights

1. This study aimed to identify the correlates of infant mortality in Ethiopia based on 2019 Ethiopia Mini Demographic and Health Survey data.
2. The mortality rate of infants was very high in the earlier age of months.
3. Infants from rural areas had higher risk of death than infants from urban areas.
4. Infants with higher birth order were more likely to die compared to those with first birth order.
5. Infants born in health facilities were more likely to die compared with those born at homes.

The infant mortality rate, defined as the risk for a live born child to die before its first birthday, is known to be one of the most sensitive and commonly used indicators of the social and economic development of a nation^1^.

Globally, about 67% of under-five deaths are accounted by the infant mortality^2^. This clearly points out that, to achieve the desired sustainable development goal target for the reduction of under-five mortality by 2030, particular emphasis needs to be given to infant mortality.

Infant mortality rate in WHO African region (52 deaths per 1000 live births) which is over six times higher than infant mortality rate in the European region (8 deaths per 1000 live births)^3^. In Ethiopia, infant mortality rate was 47 deaths per 1000 live births in 2019. The infant mortality rate has large share among under-five mortality in Ethiopia^4^.

Infant mortality is correlated with the resources accessible to mothers. For instance, the lack of convenient access to clean drinking and sanitary water leads the women to walk considerable distances while carrying pitchers of water. They may find it simple to use electric washing machines and get drinking and sanitary water, which could enhance their health and allow them to land better employment and devote more money to their kids’ health^5^,^6^. Infant mortality drops considerably with a rise in household income^7^. The main contributing factor to the high infant mortality rate in developing countries is a lack of mother education^8^, and improving maternal literacy levels may help reduce infant mortality rates^9^.

Previous study identified different correlates of infant mortality like sex of child, breastfeeding status, size of child at birth, place of delivery, mother’s age at first birth, child’s birth order, type of birth, mother’s age, religion, maternal anaemia, maternal HIV, wealth index, mother’s educational level, diarrhoea, family size, residence, mother’s marital status, pregnancy type, region, mother’s educational level, ANC visit, source of drinking water, toilet facility, sex of household^10^–^29^.

Understanding and identifying the correlates of infant mortality is important for strategy planners to set effective interventions to reduce infant mortality, which could help to attain the sustainable development goal goal by 2030. Therefore, this study aimed to explore the correlates of infant mortality taking into consideration various demographic and socioeconomic factors based on the 2019 Ethiopia Demographic and Health Survey (2019 EMDHS) data.

## Methods

### Study area settings

This study was conducted in Ethiopia. Ethiopia is the country in East Africa bordered by Sudan and south Sudan in the west, Somalia and Djibouti in the east, Eritrea in the north and Kenya in the south. The country covers 1 112 000 square kilometres. The country has two City administrations, namely Addis Ababa and Dire Dawa, and nine administrative regions, namely Tigray, Amhara, Afar, Oromia, SNNPR, Somalia, Harari, Gambela and Benshangul-Gumuz when the 2019 EMDHS data were collected.

### Data source and study design

The data for this study were obtained from the 2019 EMDHS which was conducted by Central Statistical Agency (CSA) under the auspices of Ministry of Health. The 2019 EMDHS is national representative cross-sectional survey. The survey was nationally conducted among the nine Regional States and the two City Administrations of the country from 21 March to 28 June 2019. A total of 8663 households were interviewed with a response rate of 99%. From the total interviewed households, all women of reproductive age fulfilling the selection criteria were eligible for the survey and selected for individual interviews^4^. From the survey data, we selected variables of the interest with the complete information.

### Variable of the study

The response variable was survival time of an infant measured in months from birth until death/censored. The independent variables were classified into two groups: demographic factors (age of mother at first birth, birth order, type of birth, sex of child, age of mother, sex of household head) and socioeconomic factors (wealth index, place of delivery, educational level of mother, place of residence and region).

### Statistical analysis

In this study, the data were analyzed by statistical software STATA 14. Survival analysis was done to identify the correlates of infant mortality. The use of survival analysis is most important when some subjects are lost to follow up or when the period of observation is finite and certain patients may not experience the event of interest over the study period. In the latter case one cannot have complete information for such individuals. These incomplete observations are referred to as being censored. In essence, censoring occurs when we have some information about individual survival time, but we do not know the survival time exactly.

The distribution of survival time is characterized by the survivorship function, the probability density function, and the hazard function. Let T be a random variable associated with the survival times, t be the specified value of the random variable T and f(t) be the underlying probability density function of the survival time T . The cumulative distribution function F(t), which represents the probability that a subject selected at random will have a survival time less than some stated value t , is given


(1) $$F(t)\;=P(T\leq t)=\int_{0}^{t}f\left(u\right)\mathrm{du},t\;\geq \;0$$

Then the survivor function, $S\left(t\right),$ can be given as


(2) S(t)=P(T≥t)=1−F(t),t≥0

From equations (1) and (2) the relationship between f(t) and S(t) can be derived as


(3) $$f\left(t\right)=\frac{\mathrm{dF}\left(t\right)}{\mathrm{dt}}=\frac{d(1-S\left(t\right))}{\mathrm{dt}}=-\frac{\mathrm{dS}\left(t\right)}{\mathrm{dt}}\;,\;t\geq 0$$

The hazard function h(t) gives the instantaneous potential for failing at time t, given that the individual has survived up to time t . In contrast to the survivor function, which focuses on failing, the hazard function focuses on not failing, that is, on the event occurring. Thus, in some sense, the hazard function can be considered as giving the opposite side of the information given by the survivor function.

The hazard function h(t)≥0 , is given as:


(4) $$h(t)\;=\;\underset{∆t\to 0}{\lim}\frac{P\left\{\mathrm{an individual fails in the time interval}\;\left(t,t+∆t\right)\;\mathrm{given survived until time}\;t\right\}}{∆t}\;=\underset{∆t\to 0}{\lim}\frac{\mathrm{Pr}\left(t\leq T<t+∆\mathrm{t\backslash T}\geq t\right)}{∆t}$$

The hazard function can be expressed in terms of the underlying probability density function and the corresponding cumulative hazard function H(t) is defined as

$\;H(t)=\;\int_{0}^{t}h(u)\mathrm{du}=\;-\ln S(t)$ .


(5) ThenS(t)=exp(−H(t))andf(t)=h(t)S(t)

## Results

### Demographic and socioeconomic characteristics of infants

A total of 5753 children were included in this study. Of the total children include, 314 of them were died before celebrating their first birthdays. Out of the total of 314 deaths, 183 (58.3%) of them occurred among male infants and remaining 131 (41.7%) of them occurred among female infants. About 236 (75.2%) infant death were from mothers aged less than 25 years, 23 (7.3%) were from mothers aged 25–34 years and 55 (17.5%) were from mothers of age above 34 years (Table 1).

**Table 1:** Demographic and socioeconomic characteristics of infants in Ethiopia

Factor	Category	Censored	Death (%)	Event percentage	Total
Sex of child	Male	2786	183 (58.3)	6.2	2969
	Female	2653	131 (41.7)	4.7	2784
Birth order of child	1st	1180	81 (25.8)	6.4	1261
	2nd–5th	3060	145 (46.2)	4.5	3205
	6th +	1199	88 (28.0)	6.8	1287
Type of birth	Single	5316	270 (86.0)	4.8	5586
	Multiple	123	44 (14.0)	26.3	167
Place of residence	Rural	1261	67 (21.3)	5.0	1328
	Urban	4178	247 (78.7)	5.6	4425
Region	Tigray	441	13 (4.1)	2.9	454
	Affar	618	34 (10.8)	5.2	652
	Amhara	490	21 (6.7)	4.1	511
	Oromia	680	39 (12.4)	5.1	719
	Somali	586	51 (16.2)	8.0	637
	Benshangul-Gumuz	487	43 (13.7)	8.1	530
	SNNPR	638	22 (7.0)	3.3	660
	Gambela	421	29 (9.2)	6.4	450
	Harari	418	29 (9.2)	6.5	447
	Addis Ababa	285	6 (1.9)	2.1	291
	Dire Dawa	375	27 (8.6)	6.7	402
Age of mother	<25	4224	236 (75.2)	5.3	4460
	25–34	409	23 (7.3)	5.3	432
	35 and above	806	55 (17.5)	6.4	861
Age of mother at first birth	<20	2666	144 (45.9)	5.1	2810
	20–24	1558	92 (29.3)	5.6	1650
	25 and above	1215	78 (24.8)	6.0	1293
Educational level of mother	No education	2972	177 (56.4)	5.6	3149
	Primary	1710	113 (36.0)	6.2	1823
	Secondary and higher	757	24 (7.6)	3.1	781
Sex of household head	Male	4343	255 (81.2)	5.5	4598
	Female	1096	59 (18.8)	5.1	1155
Wealth index	Poor	2782	176 (56.1)	5.9	2958
	Middle	763	42 (13.4)	5.2	805
	Rich	1894	96 (30.6)	4.8	1990
Place of delivery	Home	2702	179 (57.0)	6.2	2881
	Health facility	2671	127 (40.4)	4.5	2798
	Other	66	8 (2.6)	10.8	74

### Survival curve estimates of infant

**Figure 1:**
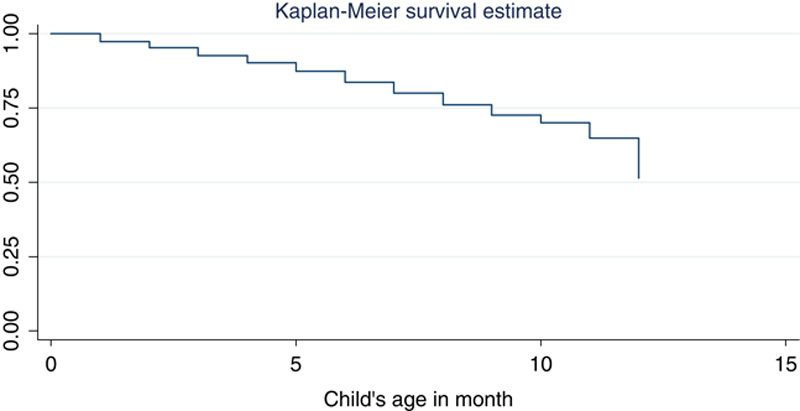
Plot of the overall estimate of Kaplan–Meier estimate of survivor function of infants.

**Figure 2:**
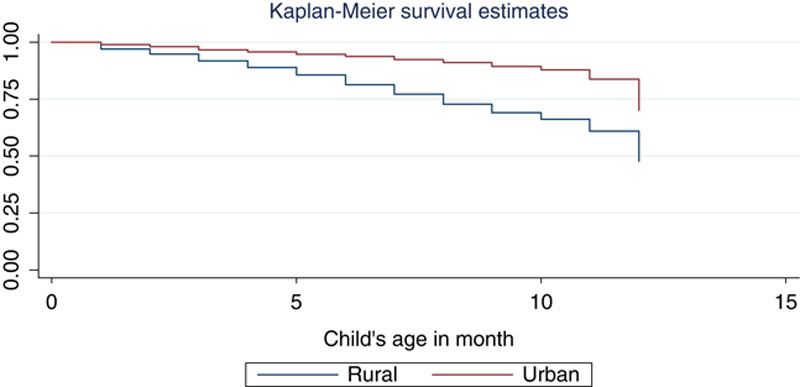
Survival curves by place of residence.

Kaplan–Meier estimator revealed that most of the deaths occurred in the earlier months of infants’ age (Fig. 1). It also revealed that urban infants had a higher survival time than rural infants (Fig. 2).

### Univariable Cox proportional hazard analysis

In univariable analysis, factors age of mother, place of residence, wealth index, birth type, birth order of child, sex of child, sex of household head and place of delivery were significant at 5% level of significance (Table 2). Factors that were significant in the univariable Cox proportional hazard analysis at 5% level of significance were included in multivariable Cox proportional hazard analysis.

**Table 2:** Result of univariable Cox proportional hazard analysis

Covariates	*P* value
Sex of child	0.000
Child’s birth order	0.016
Type of birth	0.000
Place of residence	0.000
Region	0.236
Age of mother	0.002
Age of mother at 1st birth	0.145
Educational level of mother	0.510
Sex of household head	0.029
Wealth index	0.000
Place of delivery	0.002

### Multivariable Cox proportional hazard analysis

The Multivariable Cox proportional hazard analysis result revealed that factors age of mother, place of residence, wealth index, birth type, birth order of child, sex of child and place of delivery were significant correlated with infant mortality at 5% level of significance (Table 3).

By considering age of mother below 25 as reference group, the hazard ratios are 0.656 (95% CI: 0.495–0.868) and 0.694 (95% CI: 0.556–0.867) for 25–34 and 35 and above, respectively. These suggest that infants born from mothers aged 25–34 years had 34.4% lower mortality rate than those infants born from mothers aged below 25 years. The 95% confidence interval indicates that the hazard rate goes to a maximum of 0.868 and a minimum of 0.495. Whereas infants born from mothers aged 35 years and above had 30.6% lower mortality rate than those born from mothers aged below 25 years. The 95% confidence interval indicates that the hazard rate goes to a maximum of 0.867 and a minimum of 0.556. On the other hand, the estimated hazard ratio of infants born from mothers aged 35 years and above compared with those born from mothers aged 25–34 years was 1.059=exp(−0.365−(−0.422)) . This suggests that infants born from mothers aged 35 years and above had 5.9% lower mortality rate than those born from mothers aged 25–34 years.

## Discussion

This study aimed to identify the correlates of infant mortality. Of the total of 5753 live births included in the study, 314 of them were died before celebrating their first birthdays. Among the total of 314 deaths, 183 (58.3%) occurred among male infants while remaining 131 (41.7%) occurred among female infants. About 75.2% infant deaths were observed among mothers aged less than 25 years, 7.3% were among mothers aged 25–34 years and 17.5% were among mothers of age above 34 years. Infant mortality rate was high in the earlier age of months. The study revealed that age of mother, place of residence, wealth index, birth order, type of birth, sex of child and place of delivery were significantly correlated with infant mortality.

This study revealed that infants born from mothers aged 35 years and above had lower risk of mortality than those born from mothers aged below 25 years. This finding is consistent with the result of study^10^,^30^,^31^. The possible justification could that the older mothers may have the experience of handling the infants better than the younger mothers.

Our study also revealed that an infant born in rural area had higher risk of death than an infant born in urban areas. This finding agrees with the results of the studies^10^,^30^,^32^. This could be due to that urban mothers are better educated and have better health facilities than rural ones. The sex of infant is also statistically significant predictor of the infancy death in our study. This study revealed that male infants had higher risk of dying compared to female infants. This result is consistent with the prior studies^10^,^33^–^37^. The possible reason might be that males are biologically weaker and more susceptible to diseases and premature death compared to females^38^.

Our study also revealed that infants born at health facilities were less likely to die before first birth day than infants born at home. This result is consistent with the previous studies^17^,^21^,^31^,^39^–^41^. This could be due to that babies born at health facilities may get better care during the delivery as it assisted by health professionals compared to those born at home. Infants with birth order of second to fifth were more likely to die as compared with infants with first birth order. This could be because of that as the birth order increases, the number of children in the household increases, which could increase the likelihood of the children dying because as the number of children increases, the share of food and care from the mother required for the children decreases^17^. This result is supported by the findings of the studies^17^,^42^,^43^.

Moreover, the study revealed that children born from the rich families were less likely to die before celebrating their first birthdays than those born from poor families. This is consistent with the study^32^,^44^,^45^. This might be because, in comparison to mothers from poor families, mothers from wealthy families may have access to better nutrition and healthcare during their pregnancies, as well as child-care facilities for their survival. Similarly, infants whose birth type was single were less likely to die before first birthday than infants whose birth type was multiple. This agrees with studies^11^,^17^,^21^,^31^,^42^,^45^–^50^. This could be because of that multiple births share foods and cares provided to them whereas the singletons use without sharing.

## Conclusion

The Kaplan–Meier survival function was used to estimate the survival time of infants. The result indicated that the mortality rate of infants were very high in the earlier age of months. The study found that age of mother, place of residence, wealth index, birth order, type of birth, sex of child and place of delivery were statistically significant in affecting the survival of the infants. Thus, health facility deliveries should be encouraged and multiple birth infants should be given special care. Furthermore, younger mothers should better care of their babies to improve the survival of infants in Ethiopia.

### Limitations of the study

As this study was secondary data analysis on 2019 EMDHS, some of variables with high number of missing values like breastfeeding status of child, and father’s educational level were not included.

## Ethical approval

The data used for this study were secondary data and publicly available and have no personal identifiers. We got permission to use the EDHS data from Measure DHS international program.

## Consent

Not applicable.

## Sources of funding

This research did not receive any funding.

## Authors contribution

K.L.A.: conceptualization, data curation, formal analysis, investigation, methodology, software, validation, visualization, writing—original draft. S.K.W.: formal analysis, investigation, methodology, software, validation, visualization, writing—review and editing. S.D.Y.: formal analysis, investigation, methodology, software, visualization, writing—review & editing. K.B.G.: data curation, formal analysis, methodology, software, visualization, writing—review and editing. G.G.M.: formal analysis, investigation, methodology, software, visualization, writing—review and editing. T.S.W.: formal analysis, investigation, methodology, software, visualization, writing—review and editing. T.B.: formal analysis, investigation, methodology, software, visualization, writing—review and editing. B.H.R.: Formal analysis, Investigation, Methodology, Software, Visualization, Writing—review and editing. A.G.: data curation, formal analysis, investigation, methodology, software, visualization, writing—review and editing. K.D.Y.: formal analysis, investigation, methodology, software, visualization, writing—review and editing.

## Conflicts of interest disclosure

Authors declare that they do not have conflict of interest.

## Data availability

All data used for the current study are available upon reasonable request from corresponding author.

## Provenance and peer review

Not commissioned, externally peer-reviewed.

## Acknowledgements

The authors are grateful to DHS Program for providing the data for the study.

## Figures and Tables

**Table 3:** Result of multivariable Cox proportional hazard analysis

						95% CI for exp(*β*)	
Covariates (Reference)	*β*	*s.e*(*β*)	Wald	*P* value	Hazard ratio	Lower	Upper
Child sex (Female)	0.223	0.080	7.877	0.005	1.250	1.070	1.461
Child’s birth order (First)			14.924	0.001			
2nd–5th	0.468	0.141	10.970	0.001	1.596	1.210	2.106
6th and above	0.091	0.124	0.544	0.461	1.095	0.860	1.396
Type of birth (Multiple)	−1.269	0.119	113.586	0.000	0.281	0.223	0.355
Place of residence (Urban)	0.393	0.167	5.542	0.019	1.481	1.068	2.053
Mother’s age (below 25)			11.463	0.003			
25–34	−0.422	0.143	8.671	0.003	0.656	0.495	0.868
35 and above	−0.365	0.113	10.381	0.001	0.694	0.556	0.867
Wealth index (Poor)			56.077	0.000			
Middle	−0.514	0.348	2.180	0.062	0.598	0.249	3.428
Rich	−0.689	0.154	13.627	0.000	0.502	0.173	0.896
Sex of household head (Female)	−0.564	0.325	3.004	0.761	0.569	0.401	2.194
Place of delivery (Home)			13.794	0.001			
Health facility	−0.558	0.153	13.335	0.000	0.573	0.424	0.772
Other	−0.350	0.231	2.304	0.129	0.705	0.448	1.107
